# Pretreatment periodontitis is predictive of a poorer prognosis after esophagectomy for esophageal cancer

**DOI:** 10.1007/s10388-024-01045-z

**Published:** 2024-02-20

**Authors:** Shu Nozaki, Yusuke Sato, Hiroshi Takano, Kyoko Nomura, Akiyuki Wakita, Jiajia Liu, Yushi Nagaki, Ryohei Sasamori, Yoshihiro Sasaki, Tsukasa Takahashi, Hidemitsu Igarashi, Yasunori Konno, Masayuki Fukuda, Yoshihiro Minamiya

**Affiliations:** 1 Esophageal Surgery, Akita University Hospital, Akita, 010-8543 Japan; 2 Department of Thoracic Surgery, Akita University Graduate School of Medicine, Akita, 010-8543 Japan; 3 Department of Dentistry and Oral Surgery, Akita University Graduate School of Medicine, Akita, 010-8543 Japan; 4 Department of Environmental Health Science and Public Health, Akita University Graduate School of Medicine, Akita, 010-8543 Japan

**Keywords:** Esophageal cancer, Esophageal squamous cell carcinoma, ESCC, Periodontitis, Oral condition, Prognosis, Toll-like receptor, TLR

## Abstract

**Background:**

Poor oral health is an independent risk factor for upper-aerodigestive tract cancers, including esophageal cancer. Several studies have investigated short-term outcomes after esophagectomy and the impact of periodontal disease, but few have examined the impact of periodontal disease on long-term outcomes. The purpose of this study was to investigate the rate of periodontitis among esophagectomy patients and the prognostic value of periodontitis and its effect on prognosis after esophagectomy.

**Methods:**

A total of 508 patients who underwent esophagectomy received oral health care from a dentist before cancer treatment at Akita University Hospital between January 2009 and December 2021. We assessed the presence and severity of the patients’ periodontitis and divided them into no-periodontitis, mild periodontitis, severe periodontitis and edentulous jaw groups. We then assessed 10-year overall survival (OS) and disease-specific survival (DSS) and determined whether periodontitis was an independent prognostic factor affecting OS and DSS.

**Results:**

We found that 101 (19.9%) patients had no periodontitis, 207 (40.8%) had mild periodontitis, 176 (34.6%) had severe periodontitis requiring tooth extraction, and 24 (4.7%) had edentulous jaw. Both OS and DSS were significantly poorer in the periodontitis than no-periodontitis group (*p* < 0.001). In detail, the edentulous jaw group had the poorest prognosis (*p* < 0.001). Multivariate analysis showed that periodontitis was an independent risk factor affecting OS and DSS.

**Conclusion:**

Esophageal cancer patients had a high prevalence of periodontitis. Moreover, the presence of periodontitis and severity of periodontitis are independent risk factors contributing to a poorer prognosis after esophagectomy.

**Supplementary Information:**

The online version contains supplementary material available at 10.1007/s10388-024-01045-z.

## Introduction

Esophageal squamous cell carcinoma (ESCC) is the most common histological type of esophageal cancer in Africa, Central South America, and Asia [1, 2]. Known risk factors for ESCC include habitual cigarette smoking, heavy alcohol consumption, hereditary inactive acetaldehyde dehydrogenase 2 (ALDH2) and a poor diet lacking green and yellow vegetables [3, 4]. More recently, it was reported that poor oral health is also an independent risk factor for upper-aerodigestive tract cancers, including ESCC [5, 6].

Poor oral health usually involves periodontitis, which reportedly correlates with a variety of systemic diseases, including diabetes, heart disease, atherosclerosis, stroke, Alzheimer’s disease, immature birth and various cancers [7–10]. We previously reported that approximately 70% of our ESCC patients have periodontal disease and about half of those patients required one or more dental extractions before treatment of their cancer [11]. In that regard, tooth loss was recently shown to be associated with a poorer prognosis in ESCC patients [12, 13].

Thanks to advances in multidisciplinary treatments, the prognosis of esophageal cancer patients has improved in recent years [14, 15]. One important treatment is esophagectomy. However, this is a highly invasive gastrointestinal surgery with a high incidence of complications [15]. Among these complications, postoperative pneumonia is commonly observed, and there have been reports suggesting that appropriate oral care can reduce its incidence [11, 16]. Perioperative oral care is therefore considered essential for safe postoperative management after esophagectomy.

In Japan, perioperative management of the oral health of patients with malignant tumors was first covered by health insurance in 2012. Nonetheless, since 2009 we have been routinely providing oral health assessment and oral care by dentists to ESCC patients prior to the start of cancer treatment. By 2021, a total of 508 patients underwent surgery after preoperative oral health evaluation at our hospital. Up to now there have been scattered reports on the relationship between periodontal disease and short-term outcomes after esophagectomy, but there have been few on the effect of periodontal disease on long-term outcomes. In the present study, therefore, we investigated the presence and severity of periodontal disease and its impact on 10-year overall survival (OS) and disease-specific survival (DSS) after esophagectomy for ESCC. We also investigated whether the presence of periodontal disease is the independent prognostic factor affecting 10-year OS.

## Materials and methods

### Patients

This study was approved by the Ethics Committee of Akita University School of Medicine (#2959, March 2023), and all experiments were performed in accordance with the Helsinki Declaration. All study participants provided informed written consent. Between January 2009 and December 2021, 563 ESCC patients received esophagectomy at Akita University Hospital. Among them, 508 patients received oral health assessment and care by a dentist before cancer treatment. For all these patients, tumor staging was based on the International Union Against Cancer Tumor-Node-Metastasis (TMN) Classification of Malignant Tumors (seventh edition) [17]. Diagnostic evaluation prior to treatment included esophagography, endoscopy, computed tomography (CT) and 18F-fluorodeoxyglucose positron-emission tomography CT. The patients’ preoperative nutritional status and respiratory function were analyzed based on clinical examination data, including %VC, FEV1.0% and albumin. Surgical invasion was assessed based on operation type, operation time and operation bleeding.

### Dental management

Dental examinations were performed by a dentist at the time of the initial visit to our hospital. In each patient, periodontitis was classified into 1 of 3 categories: no periodontitis, mild periodontitis or severe periodontitis. Severe periodontitis was accompanied by the need for dental extraction, the criteria for which was (i) Miller’s tooth mobility classification class 3: > 1 mm horizontal + vertical mobility, (ii) > 4 mm periodontal pocket with bleeding or purulent discharge, (iii) severe caries of the teeth with periodontitis, and (iv) teeth with a periapical abscess or cyst [18]. An edentulous jaw was considered to have lost teeth already and was included as periodontal disease. For slight periodontitis, removal of plaque and dental calculus was performed. For dental caries without criteria for tooth extraction, removal of infected tissue and tooth crown restoration were performed. The dentist also explained the importance of oral care and how to perform self-care to all patients.

### Treatment strategy

For all patients, staging and treatment strategies were defined at a conference attended by radiologists, physicians and surgeons. In general, patients with locally advanced or node-positive tumors underwent neoadjuvant chemoradiotherapy (NACRT). Briefly, the radiotherapy consisted of 41.4 Gy in 23 fractions. The chemotherapy consisted of a protracted infusion of 5-fluorouracil (800 mg/m^2^/day) on days 1–5 combined with cisplatin or nedaplatin (80 mg/m^2^/day) on day 1. This chemotherapy protocol was repeated twice with 3- to 4-weeks interval in between [19, 20]. Our standard operative procedure was transthoracic or thoracoscopic esophagectomy with three-field lymphadenectomy of the mediastinal (involving the periesophageal region and areas around the trachea and bilateral main bronchus), abdominal (involving the perigastric region and areas around the celiac axis) and cervical (involving the bilateral periesophageal region and supraclavicular region) lymph nodes. Reconstruction commonly involved inserting a gastric tube or pedicled colon [21]. A jejunostomy feeding tube was used for patients who needed reconstruction with a pedicled colon graft or were in poor general condition.

### Statistical analyses

To test for statistical differences between the periodontitis and no-periodontitis groups, the Wilcoxon test was used for continuous variables and the chi-square and Fisher’s exact tests were used for categorical variables. Outcomes included DSS and OS, which were respectively calculated as the time from the date of esophagectomy to death from esophageal cancer or from any cause. Patients known to be alive or lost to follow-up on the date of last contact were censored in OS. Patients who passed away with other than esophageal cancer were also censored in DSS. Patients The Kaplan–Meier method was used to construct OS and DSS curves, which were compared using the log-rank test. To investigate the impact of periodontitis and to identify independent prognostic factors affecting DSS and OS, we applied a Cox proportional hazard model to calculate the hazard ratios (HRs) and 95% confidence intervals (CIs). After entering all potential predictors for DSS and OS, we ran backward selection based on type III score chi‐square test statistics to identify the most suitable models. We then checked whether the selected variables satisfied the proportional hazard assumption. To minimize the influence of a short observation period, we run sensitivity analyses by eliminating those who were followed 6 months or shorter.

All statistical analyses were performed using JMP Pro15.0 (SAS Institute, Cary, NC, USA), and two-sided *p* values < 0.05 were considered statistically significant.

## Results

**Fig. 1:**
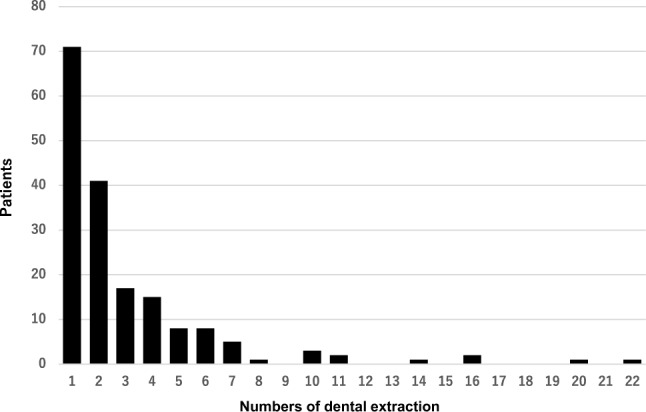
Histogram showing the distribution of the total numbers of teeth extracted from 176 patients diagnosed with severe periodontitis requiring tooth extraction. From most of these patients 1 or 2 teeth were extracted, though the maximum was 22

In the 508 cases, the histological types were SCC in 460 patients (90.6%), adenocarcinoma in 36 patients (7.1%) and other cancer types in 12 patients (2.3%). Based on dental examinations, the oral health profile of the 508 study participants was as follows: 101 (19.9%) had no periodontitis, 207 (40.8%) had mild periodontitis, 176 (34.6%) had severe periodontitis requiring tooth extractions, and 24 (4.7%) had edentulous jaws. The numbers of teeth extracted from patients with severe periodontitis are shown in Fig. 1. While most of these patients required extraction of 1 or 2 teeth, some required extraction of as many as 22 teeth.

The clinicopathological features of the no-periodontitis and periodontitis groups are summarized in Table 1. There were no significant differences with respect to sex, age at surgery, Brinkman index, alcohol consumption, tumor location, histologic type, cN, clinical stage (UICC7), pN, pathological stage (UICC7), albumin, %VC, FEV1.0%, operation type, operation time or operation bleeding. On the other hand, the periodontitis group had significantly higher smoking habitation (*p* = 0.030), higher cT (*p* = 0.033), higher neoadjuvant therapy rate (*p* = 0.004), and higher pT (*p* = 0.026).

**Table 1:** Clinicopathological features of 508 patients

Characteristics	No-periodontitis	Periodontitis	*p*
	(*n* = 101)	(*n* = 407)	
	*N* (%)	*N* (%)	
Sex			0.669
Female	14 (13.9%)	50 (12.3%)	
Male	87 (86.1%)	357 (87.7%)	
Median age at surgery range	67 (38–82)	66 (35–85)	0.11
Smoking habits			0.030*
Current	39 (38.6%)	197 (48.4%)	
Past	38 (37.6%)	138 (33.9%)	
Never	18 (17.8%)	66 (16.2%)	
Unknown	6 (6.0%)	6 (1.5%)	
Brinkman Index			0.334
0	18 (17.8%)	66 (16.2%)	
Less 400	16 (15.9%)	56 (13.8%)	
More 400	60 (59.4%)	271 (66.6%)	
Unknown	7 (6.9%)	14 (3.4%)	
Alcohol consumption			0.215
Habitual	74 (73.3%)	329 (80.8%)	
Opportunity	6 (5.9%)	26 (6.4%)	
Never	16 (15.8%)	42 (10.3%)	
Unknown	5 (5.0%)	10 (2.5%)	
Tumor location			0.189
Ce	2 (2.0%)	11 (2.8%)	
Ut	16 (15.8%)	52 (12.8%)	
Mt	54 (53.5%)	180 (44.2%)	
Lt	18 (17.8%)	119 (29.2%)	
Ae	11 (10.9%)	45 (11.0%)	
Histologic type			0.594
SCC	93 (92.1%)	367 (90.2%)	
Adeno	5 (5.0%)	31 (7.6%)	
Others	3 (2.9%)	9 (2.2%)	
Tumor invasion (cT)			0.033*
1	39 (38.6%)	120 (29.5%)	
2	14 (13.9%)	32 (7.9%)	
3	45 (44.5%)	244 (59.9%)	
4a + 4b	3 (3.0%)	11 (2.7%)	
Lymph node metastasis (cN)			0.056
0	58 (57.4%)	171 (42.0%)	
1	31 (30.7%)	152 (37.4%)	
2	9 (8.9%)	66 (16.2%)	
3	3 (3.0%)	14 (3.4%)	
4 (M1 lymph +)	0	4 (1.0%)	
Clinical Stage (UICC7)			0.053
1A + 1B	43 (42.6%)	116 (28.5%)	
2A + 2B	23 (22.8%)	85 (20.9%)	
3A + 3B + 3C	35 (34.6%)	200 (49.1%)	
4	0	6 (1.5%)	
Neoadjuvant therapy			0.004*
CRT	28 (27.7%)	174 (42.7%)	
Chemo	3 (3.0%)	31 (7.6%)	
RT	0	1(0.3)	
ESD	8 (7.9%)	31 (7.6%)	
Salvage	4 (4.0%)	24 (5.9%)	
Nothing	58 (57.4%)	146 (35.9)	
Tumor invasion(pT)			0.026*
0	14 (13.9%)	46 (11.3%)	
1	50 (49.5%)	155 (38.1%)	
2	11 (10.9%)	34 (8.3%)	
3	25 (24.7%)	146 (35.9%)	
4a + 4b	1 (1.0%)	26 (6.4%)	
Lymph node metastasis(pN)			0.268
0	61 (60.4%)	228 (56.0%)	
1	31 (30.7%)	111 (27.3%)	
2	7 (6.9%)	49 (12.0%)	
3	2 (2.0%)	19 (4.7%)	
Pathological stage (UICC7)			0.115
0	10 (9.9%)	38 (9.3%)	
1A + 1B	39 (38.6%)	121 (29.7%)	
2A + 2B	34 (33.7%)	122 (30.0%)	
3A + 3B + 3C	18 (17.8%)	125 (30.7%)	
4	0	1 (0.3%)	
Albumin(mg/dl)			0.058
Normal	62 (61.4%)	207 (50.9%)	
Less than 4.0	39 (38.6%)	200 (49.1%)	
%VC			0.463
Over 80%	97 (96.0%)	377 (92.6%)	
Under 80%	3 (3.0%)	24 (5.9%)	
Unknown	1 (1.0%)	6 (1.5%)	
FEV1.0%			0.797
Over 70%	77 (76.2%)	319 (78.4%)	
Under 70%	23 (22.8%)	82 (20.1%)	
Unknown	1(1.0%)	6 (1.5%)	
Operation type			0.363
Transthoracic	51 (50.5%)	226 (55.5%)	
Thoracoscopic	50 (49.5%)	181 (44.5%)	
Operation time			0.234
Over 538 min	56 (55.4%)	252 (61.9%)	
Under 538 min	45 (44.6%)	155 (38.1%)	
Operation bleeding			0.124
Over 692 ml	24 (23.8%)	105 (25.8%)	
Under 692 ml	76 (75.2%)	302 (74.2%)	
Unknown	1 (1.0%)	0	

* Considered significant

**Fig. 2:**
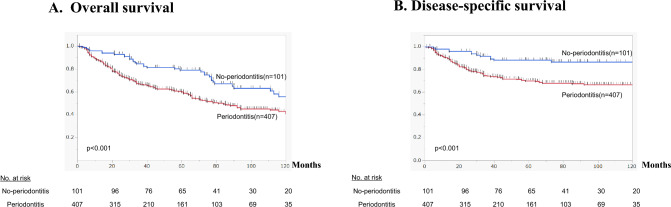
Kaplan–Meier curves illustrating the association between periodontitis (with or without) and OS (**A**) and DSS (**B**) in patients after esophagectomy

**Fig. 3:**
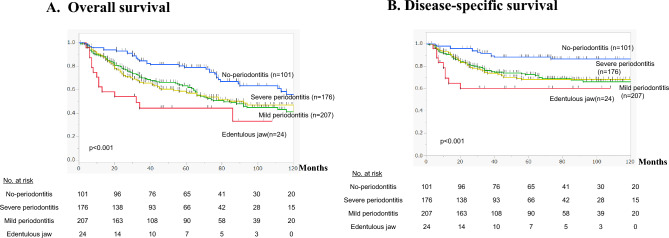
Kaplan–Meier curves illustrating the association between the degree of periodontitis (none, mild, severe or edentulous jaw) and OS (**A**) and DSS (**B**) in patients after esophagectomy

Kaplan–Meier curves comparing survival in the periodontitis and no-periodontitis groups showed that both OS (Fig. 2A) and DSS (Fig. 2B**)** were significantly higher in the no-periodontitis group (*p* < 0.001). When patients in the periodontitis group were subdivided based on the severity of their periodontitis (Table 2), Kaplan–Meier curves showed OS to be significantly higher in the no-periodontitis than in either the mild or severe periodontitis subgroup (*p* < 0.001) (Fig. 3A**)**. No significant difference was detected between the mild and severe periodontal subgroups. Notably, the edentulous jaw group had the poorest prognosis. Comparison of DSS between the no-periodontitis and three periodontitis subgroups yielded similar results (Fig. 3B).

**Table 2:** Clinicopathological features of 508 patients subdivided based on the severity of their periodontitis

Characteristics	No-periodontitis	Mild periodontitis	Severe periodontitis	Edentulous jaw	*p*
	*n* = 101	*n* = 207	*n* = 176	*n* = 24	
	(19.90%)	(40.80%)	(34.60%)	(4.70%)	
Sex					0.701
Female	14 (13.9%)	29 (14.0%)	18 (10.2%)	3 (12.5%)	
Male	87 (86.1%)	178 (86.0%)	158 (89.8%)	21 (87.5%)	
Median age at surgery range	67 (38–82)	66 (35–85)	65 (39–85)	70 (60–82)	0.001*
Smoking habits					0.048*
Current	39 (38.6%)	88 (42.5%)	95 (54.0%)	14 (58.3%)	
Past	38 (37.6%)	74 (35.8%)	58 (33.0%)	6 (25.0%)	
Never	18 (17.8%)	41 (19.8%)	21 (11.9%)	4 (16.7%)	
Unknown	6 (6.0%)	4 (1.9%)	2 (1.1%)	0	
Brinkman Index					0.163
0	18 (17.8%)	40 (19.3%)	22 (12.5%)	4 (16.7%)	
Less 400	16 (15.9%)	32 (15.5%)	23 (13.1%)	1 (4.2%)	
More400	60 (59.4%)	125 (60.4%)	127 (72.1%)	19 (79.1%)	
Unknown	7 (6.9%)	10 (4.8%)	4 (2.3%)	0	
Alcohol consumption					0.751
Habitual	74 (73.3%)	165 (79.7%)	144 (81.8%)	20 (83.4%)	
Opportunity	6 (5.9%)	13 (6.3%)	11 (6.3%)	2 (8.3%)	
Never	16 (15.8%)	24 (11.6%)	16 (9.1%)	2 (8.3%)	
Unknown	5 (5.0%)	5 (2.4%)	5 (2.8%)	0	
Tumor location					0.020*
Ce	2 (2.0%)	2 (1.0%)	6 (3.4%)	3 (12.5%)	
Ut	16 (15.8%)	22 (10.6%)	27 (15.3%)	3 (12.5%)	
Mt	54 (53.5%)	101 (48.8%)	68 (38.7%)	11 (45.8%)	
Lt	18 (17.8%)	58 (28.0%)	57 (32.4%)	4 (16.7%)	
Ae	11 (10.9%)	24 (11.6%)	18 (10.2%)	3 (12.5%)	
Histologic type					0.626
SCC	93 (92.1%)	189 (91.3%)	156 (88.6%)	22 (91.6%)	
Adeno	5 (5.0%)	16 (7.7%)	14 (8.0%)	1 (4.2%)	
Others	3 (2.9%)	2 (1.0%)	6 (3.4%)	1 (4.2%)	
Tumor invasion (cT)					0.203
1	39 (38.6%)	63 (30.4%)	51 (29.0%)	6 (25.0%)	
2	14 (13.9%)	13 (6.2%)	15 (8.5%)	4 (16.7%)	
3	45 (44.5%)	125 (60.4%)	106 (60.2%)	13 (54.2%)	
4a + 4b	3 (3.0%)	6 (3.0%)	4 (2.3%)	1 (4.1%)	
Lymph node metastasis (cN)					0.083
0	58 (57.4%)	87 (42.0%)	70 (39.8%)	14 (58.3%)	
1	31 (30.7%)	79 (38.2%)	64 (36.4%)	9 (37.5%)	
2	9 (8.9%)	29 (14.0%)	36 (20.4%)	1 (4.2%)	
3	3 (3.0%)	10 (4.8%)	4 (2.3%)	0	
4 (M1 lymph +)	0	2 (1.0%)	2 (1.1%)	0	
Clinical Stage (UICC7)					0.169
1A + 1B	43 (42.6%)	60 (29.0%)	48 (27.3%)	8 (33.3%)	
2A + 2B	23 (22.8%)	41 (19.8%)	37 (21.0%)	7 (29.2%)	
3A + 3B + 3C	35 (34.6%)	103 (49.8%)	88 (50.0%)	9 (37.5%)	
4	0	3 (1.4%)	3 (1.7%)	0	
Neoadjuvant therapy					0.002*
CRT	28 (27.7%)	88 (42.5%)	82 (46.6%)	4 (16.7%)	
Chemo	3 (3.0%)	17 (8.2%)	13 (7.4%)	1 (4.2%)	
RT	0	0	1 (0.6%)	0	
ESD	8 (7.9%)	19 (9.2%)	11 (6.2%)	1 (4.2%)	
Salvage	4 (4.0%)	9 (4.4%)	11 (6.2%)	4 (16.7%)	
Nothing	58 (57.4%)	74 (35.7%)	58 (33.0%)	14 (58.2%)	
Tumor invasion (pT)					0.163
0	14 (13.9%)	24 (11.6%)	21 (11.9%)	1 (4.2%)	
1	50 (49.5%)	80 (38.6%)	68 (38.6%)	7 (29.1%)	
2	11 (10.9%)	17 (8.2%)	13 (7.4%)	4 (16.7%)	
3	25 (24.7%)	73 (35.3%)	64 (36.4%)	9 (37.5%)	
4a + 4b	1 (1.0%)	13 (6.3%)	10 (5.7%)	3 (12.5%)	
Lymph node metastasis (pN)					0.177
0	61 (60.4%)	105 (50.7%)	110 (62.5%)	13 (54.2%)	
1	31 (30.7%)	62 (30.0%)	40 (22.7%)	9 (37.5%)	
2	7 (6.9%)	30 (14.5%)	17 (9.7%)	2 (8.3%)	
3	2 (2.0%)	10 (4.8%)	9 (5.1%)	0	
Pathological stage (UICC7)					0.342
0	10 (9.9%)	17 (8.2%)	20 (11.4%)	1 (4.2%)	
1A + 1B	39 (38.6%)	58 (28.0%)	56 (31.8%)	7 (29.2%)	
2A + 2B	34 (33.7%)	63 (30.4%)	53 (30.1%)	6 (25.0%)	
3A + 3B + 3C	18 (17.8%)	68 (32.9%)	47 (26.7%)	10 (41.6%)	
4	0	1 (0.5%)	0	0	
Albumin (mg/dl)					0.176
Normal	62 (61.4%)	111 (53.6%)	85 (48.3%)	11 (45.8%)	
Less than 4.0	39 (38.6%)	96 (46.4%)	91 (51.7%)	13 (54.2%)	
%VC					0.031*
Over 80%	97 (96.0%)	194 (93.7%)	164 (93.2%)	19 (79.2%)	
Under 80%	3 (3.0%)	9 (4.4%)	10 (5.7%)	5 (20.8%)	
Unknown	1 (1.0%)	4 (1.9%)	2 (1.1%)	0	
FEV1.0%					0.42
Over 70%	77 (76.2%)	162 (78.3%)	142 (80.7%)	15 (62.5%)	
Under 70%	23 (22.8%)	41 (19.8%)	32 (18.2%)	9 (37.5%)	
Unknown	1 (1.0%)	4 (1.9%)	2 (1.1%)	0	
Operation type					0.82
Transthoracic	51 (50.5%)	114 (55.1%)	98 (55.7%)	14 (58.3%)	
Thoracoscopic	50 (49.5%)	93 (44.9%)	78 (44.3%)	10 (41.7%)	
Operation time					0.13
Over 538 min	56 (55.4%)	119 (57.5%)	119 (67.6%)	14 (58.3%)	
Under 538 min	45 (44.6%)	88 (42.5%)	57 (32.4%)	10 (41.7%)	
Operation bleeding					0.541
Over 692 ml	24 (23.8%)	51 (24.6%)	49 (27.8%)	5 (20.8%)	
Under 692 ml	76 (75.2%)	156 (75.4%)	127 (72.2%)	19 (79.2%)	
Unknown	1 (1.0%)	0	0	0	

* Considered significant

Univariate analyses showed that periodontitis (periodontitis vs no-periodontitis), sex (male vs female), age at surgery (65 over vs under 65), tumor invasion (cT; T3-4 vs T1-2), lymph node metastasis (cN; N + vs N0), clinical stage (UICC’7; over 3A vs under 2B), tumor invasion (pT; T3-4 vs T1-2), lymph node metastasis (N + vs N0), pathological stage (UICC7; over 3A vs under 2B), %VC (under 80% vs other) and operation type (transthoracic vs thoracoscopic) were all significant prognostic factors significantly affecting OS. Among these factors, multivariate analysis showed that periodontitis, sex, age at surgery, tumor invasion (cT), pathological stage (UICC7), and %VC were independent risk factors affecting 10-year OS. The HR for the effect of periodontitis on OS was 1.525 (Table 3) and on DSS was 2.040 (Table 4). Moreover, the severity of periodontitis was also independent risk factors affecting OS (Table 5). The HR for the effect of the mild periodontitis, severe periodontitis and edentulous jaw group on OS were 1.424, 1.592 and 2.083, respectively.

**Table 3:** Univariate and multivariable analyses of 10-year OS

10-year overall survival	Univariate Cox PH Model			Multivariable Cox PH Model		
Variable	*p*	Hazard Ratio	95% CI	*p*	Hazard Ratio	95% CI
Periodontitis (periodontitis vs no periodontitis)	< 0.001*	1.887	1.301–2.735	0.030*	1.525	1.042–2.232
Sex (male vs female)	0.007*	1.975	1.204–3.241	0.003*	2.151	1.305–3.546
Age at Surgery (over 65 vs under 65)	0.024*	1.365	1.043–1.787	0.031*	1.359	1.029–1.794
Brinkman Index (over 400 vs other)	0.058	1.319	0.991–1.756			
Alcohol consumption (Habitual vs other)	0.148	1.292	0.914–1.827			
Tumor invasion (cT) (T3-4 vs T1-2)	< 0.001*	2.535	1.877–3.423	< 0.00*1	2.252	1.392–3.642
Lymph node metastasis (cN) (N + vs N0)	< 0.001*	1.770	1.342–2.335	0.780	1.089	0.599–1.979
Clinical Stage (UICC7) (over 3A vs under 2B)	< 0.001*	1.883	1.439–2.464	0.594	0.833	0.426–1.629
Neoadjuvant therapy (none vs with)	0.826	1.030	0.788–1.349			
Tumor invasion (pT) (T3-4 vs T1-2)	< 0.001*	2.220	1.701–2.897	0.184	0.763	0.511–1.137
Lymph node metastasis (pN) (N + vs N0)	< 0.001*	1.958	1.500–2.556	0.683	0.917	0.604–1.392
Pathological stage (UICC7) (over 3A vs under 2B)	< 0.001*	3.205	2.449–4.195	< 0.001*	3.034	1.862–4.943
Albumin (less than 4.0 mg/dl vs normal)	0.054	1.302	0.996–1.702			
%VC (under 80% vs other)	0.015*	1.881	1.130–3.132	0.006*	2.080	1.229–3.522
FEV1.0% (other vs under 70% other)	0.470	0.882	0.628–1.239			
Operation type (Transthoracic vs Thoracoscopic)	< 0.001*	1.745	1.304–2.335	0.164	1.249	0.913–1.709
Operation time (over 538 min vs under 538 min)	0.750	0.957	0.729–1.255			
Operation bleeding (over 692 ml vs other)	0.299	1.165	0.873–1.554			

* Considered significant

**Table 4:** Univariate and multivariable analyses of 10-year DSS

10-year Disease-specific survival	Univariate Cox PH Model			Multivariable Cox PH Model		
Variable	*p*	Hazard Ratio	95% CI	*p*	Hazard Ratio	95% CI
Periodontitis (periodontitis vs no periodontitis)	< 0.001*	2.878	1.586–5.222	0.021*	2.040	1.114–3.737
Sex (male vs female)	0.022*	2.312	1.129–4.731	0.023*	2.305	1.112–4.735
Age at surgery (over 65 vs under 65)	0.767	0.948	0.668–1.347	^*^		
Brinkman index (over 400 vs other)	0.137	1.339	0.911–1.966			
Alcohol consumption (Habitual vs other)	0.238	1.327	0.830–2.121			
Tumor invasion (cT) (T3-4 vs T1-2)	< 0.001*	4.698	2.880–7.663	0.007*	3.012	1.354–6.701
Lymph node metastasis (cN) (N + vs N0)	< 0.001*	3.294	2.163–5.018	0.068	2.346	0.941–5.849
Clinical stage (UICC7) (over 3A vs under 2B)	< 0.001*	3.212	2.180–4.733	0.181	0.505	0.185–1.375
Neoadjuvant therapy (without vs with)	0.092	1.376	0.950–1.995			
Tumor invasion (pT) (T3-4 vs T1-2)	< 0.001*	3.897	2.686–5.655	0.999	1.000	0.579–1.727
Lymph node metastasis (pN) (N + vs N0)	< 0.001*	3.877	2.631–5.713	0.777	1.092	0.594–2.007
Pathological stage (UICC7) (over 3A vs under 2B)	< 0.001*	6.524	4.517–9.422	< 0.001*	4.062	2.064–7.993
Albumin (less than 4.0 mg/dl vs normal)	0.625	1.092	0.767–1.555			
%VC (under 80% vs over 80%)	0.258	1.512	0.738–3.095			
FEV1.0% (under 70% vs over 70%)	0.416	0.826	0.521–1.309			
Operation type (Transthoracic vs Thoracoscopic)	< 0.001*	1.919	1.306–2.818	0.827	1.046	0.697–1.571
Operation time (over 538 min vs under 538 min)	0.601	0.910	0.639–1.296			
Operation bleeding (over 692 ml vs other)	0.533	1.131	0.769–1.666			

* Considered significant

**Table 5:** Univariate and multivariable analyses of 10-year OS including the severity of periodontitis

10-year overall survival	Univariate Cox PH Model			Multivariable Cox PH Model		
Variable	*p*	Hazard Ratio	95% CI	*p*	Hazard Ratio	95% CI
Periodontitis		1.000				
(mild vs no periodontitis)	0.004*	1.800	1.209–2.680	0.088	1.424	0.948–2.139
(severe vs no periodontitis)	0.003*	1.860	1.238–2.796	0.030*	1.592	1.047–2.420
(edentulous jaws vs no periodontitis)	< 0.001*	3.228	1.723–6.048	0.027*	2.083	1.087–3.990
Sex (male vs female)	0.007*	1.975	1.204–3.241	0.003*	2.156	1.307–3.556
Age at Surgery (over 65 vs under 65)	0.024*	1.365	1.043–1.787	0.046*	1.332	1.005–1.767
Brinkman Index (over 400 vs other)	0.058	1.319	0.991–1.756			
Alcohol consumption (Habitual vs other)	0.148	1.292	0.913–1.827			
Tumor invasion (cT) (T3-4 vs T1-2)	< 0.001*	2.535	1.877–3.423	0.001*	2.247	1.389–3.637
Lymph node metastasis (cN) (N + vs N0)	< 0.001*	1.770	1.342–2.335	0.751	1.102	0.605–2.005
Clinical Stage (UICC7) (over 3A vs under 2B)	< 0.001*	1.883	1.439–2.464	0.586	0.830	0.424–1.624
Neoadjuvant therapy (none vs with)	0.826	1.030	0.788–1.349			
Tumor invasion (pT) (T3-4 vs T1-2)	< 0.001*	2.220	1.701–2.897	0.196	0.768	0.514–1.146
Lymph node metastasis (pN) (N + vs N0)	< 0.001*	1.958	1.500–2.556	0.723	0.927	0.608–1.412
Pathological stage (UICC7) (over 3A vs under 2B)	< 0.001*	3.205	2.449–4.195	< 0.001*	2.995	1.835–4.887
Albumin (less than 4.0 mg/dl vs normal)	0.054	1.302	0.996–1.702			
%VC (under 80% vs other)	0.015*	1.881	1.130–3.132	0.012*	1.979	1.162–3.373
FEV1.0% (other vs under 70% other)	0.470	0.882	0.628–1.239			
Operation type (Transthoracic vs Thoracoscopic)	< 0.001*	1.745	1.304–2.335	0.188	1.235	0.902–1.692
Operation time (over 538 min vs under 538 min)	0.750	0.975	0.729–1.255			
Operation bleeding (over 692 ml vs other)	0.299	1.165	0.873–1.554			

* Considered significant

Sensitivity analyses by eliminating those who were followed 6 months or shorter confirmed significance of periodontitis associated with both of OS and DSS (Supplementary Table 1,2).

## Discussion

The present study shows that more than 80% of our patients who underwent esophagectomy for esophageal cancer had periodontitis and that 35% of those patients required tooth extraction. We also showed that periodontitis, irrespective of severity, was an independent risk factor reducing OS and that edentulous jaw patients had the poorest prognoses.

We did not detect a difference in OS between patients with severe and mild periodontitis, but edentulous jaw patients had the poorest prognosis. We suggest that the oral environment is improved by tooth extraction and that professional oral care by a dentist was effective. On the other hand, patients with edentulous jaws have already lost many teeth and are the most severely affected by periodontitis. Consistent with our findings, an earlier study reported that patients who required more than 13 tooth extractions had the poorest prognosis [13]. However, the median age of this group was 5 years older than the other groups (median age: 67, 65, 65 and 70, respectively).

Several studies have reported that periodontal disease increases the risk not only for esophageal cancer but also for a variety of other cancers, including gastric, colorectal, pancreatic, lung, kidney and hematologic cancers [22–25]. It also appears to be closely associated with such life-threatening diseases as heart disease, atherosclerosis, stroke and aspiration pneumonia [8, 9, 26]. Notably, we detected a significant difference in DSS between the periodontitis and no-periodontitis groups, suggesting that periodontitis plays a key role specifically in the development, progression and recurrence of esophageal cancer.

The molecular mechanisms underlying the poorer prognosis among ESCC patients with periodontitis remain unclear. However, it is known that periodontitis is most often caused by the gram-negative bacteria *Porphyromonas gingivalis*, *Tannerella forsythia* and *Treponema denticola*, which together are categorized as the “red complex.” For example, *P. gingivalis* is detected in about 80% of patients with chronic periodontitis [27]. These gram-negative bacteria release lipopolysaccharides (LPS), which are associated with the progression and metastasis of ESCC cells [28, 29]. LPS is recognized by Toll-like receptor 4 (TLR4), high expression of which was previously shown to be an independent factor contributing to a poorer prognosis after esophagectomy [30, 31]. Accordingly, periodontitis may contribute to tumor progression and development by stimulating TLR4, which in turn leads to a poorer OS and DSS.

On the other hand, we recently reported that high expression of TLR6, which recognizes peptidoglycan (PGN) released from gram-positive bacteria in cancer tissue, is predictive of a significantly better prognosis after esophagectomy [32]. So-called “beneficial bacteria” in the gut bacterial flora, such as members of the genus *Lactobacillus* as well as *Bacillus subtilis* and several butyrate-producing strains are all gram-positive bacteria [33–35]. More importantly, normal inhabitants in oral flora and categorized “early colonizer”, including *Streptococcus mitis*, *Streptococcus oralis* and *Streptococcus salivarius* are all gram-positive bacteria [36]. These gram-positive strains release PGN, which we previously showed to stimulate TLR6 expressed on the surface of ESCC lines and to suppress the proliferation of these cells [32]. This suggests that a predominance of “beneficial” gram-positive bacteria in the oral environment is an important factor in improving prognosis after esophagectomy.

In our previous study, microorganisms most frequently encountered in sputum cultures from the patients with postoperative pneumonia after esophagectomy were *MRSA (Methicillin-resistant Staphylococcus aureus)*, *MRSE (Methicillin-resistant Staphylococcus epidermidis)* and *candida albicans* [11]. However, periodontal gram-negative bacteria were anaerobic and categorized as oral bacteria, consequently not detected in our study methods. Another previous study showed that anaerobic oral bacteria were more frequently detected in older patients with aspiration pneumonia than previously believed [37]. Therefore, there is a possibility that periodontal gram-negative bacteria cause postoperative pneumonia in patients with loss of swallowing function after esophagectomy.

Accumulating evidences have shown that postoperative complications are correlated with poorer survival after esophagectomy [38, 39]. Among those evidences, Yuda M, et al. reported that the presence of allopatric bacteria in the oropharynx was an independent risk factor for postoperative pneumonia and correlated with significantly poorer survival [39]. Though the severity of periodontal disease of patients was not mentioned in that study, this result suggests that patients with poor oral condition are predictive of both the occurrence of postoperative pneumonia and poor prognosis after esophagectomy. Taken together, we can speculate that postoperative complications are correlated with poorer survival after esophagectomy, but those patients have poor oral conditions at the bottom.

Based on this idea and previous our findings, we started a specified clinical trial; Improvement of oral environment with toothpaste and mouse wash containing *Lactobacillus* and the incident rate of pneumonia after esophagectomy for esophageal cancer patients (LacPEC study, jRCTs021230010) in June 2023. Those toothpaste and mouse wash containing *Lactobacillus*, “beneficial” gram-positive bacteria, are already commercially available in Japan. It was already proved that this *Lactobacillus* significantly reduced periodontal gram-negative bacteria and *candida albican*s but not affected normal inhabitants in oral flora, including *Streptococcus mitis*, *Streptococcus oralis*. The results of this specified clinical trial may prove the benefit of the predominance of “beneficial” gram-positive bacteria in the oral environment before esophagectomy for preventing postoperative complications, and consequently for better survival.

This study has several limitations. First, there was selection bias due to this being a retrospective, single-center study. Second, the presence or absence and degree of periodontitis were determined by the dentist but could not be expressed in an objective measure. Third, only one evaluation of oral health was performed before the start of treatment, and there were no follow-up data on postoperative oral health. Fourth, there was no preoperative analysis of the oral microbiota in these patients. It would have been ideal if we could have tracked the degree to which periodontitis was improved by the dentist's intervention, the degree to which the patients were able to perform oral care by themselves after discharge from the hospital and how the oral microbiota changed with oral care. In the future, it will be important to establish a system to evaluate changes in the oral environment of patients after esophagectomy and its impact on patient outcome. Fifth, there were 289 censored cases although among these, there were 53 who were alive or did not have a recurrence that should be discriminated from the usual “drop-out”. After subtracting 53 from 289, there were 236 drop-out which consisted 46% of the total numbers.

In summary, patients who underwent esophagectomy had a high prevalence of periodontitis, and about half of the periodontally ill patients had severe periodontitis requiring tooth extraction. Moreover, the presence of periodontitis and severity of periodontitis are independent risk factors contributing to a poorer prognosis after esophagectomy.

### Supplementary Information

Below is the link to the electronic supplementary material.

Supplementary file1 (DOCX 22 KB)

Supplementary file2 (DOCX 22 KB)

## Abbreviations

ESCC: Esophageal squamous cell carcinoma
ALDH2: Acetaldehyde dehydrogenase 2
LPS: Lipopolysaccharide
TLR4: Toll-like receptor 4
TLR6: Toll-like receptor 6
