# Supplementary material for: Pretreatment periodontitis is predictive of a poorer prognosis after esophagectomy for esophageal cancer
Source: Esophagus. 2024 Feb 20;21(2):120–30. doi: 10.1007/s10388-024-01045-z (PMC10957679; doi:10.1007/s10388-024-01045-z)
Supplement: Supplementary file 1 — Supplementary file1 (DOCX 22 KB) [file 10388_2024_1045_MOESM1_ESM.docx]

Supplementary Table 1

| 10-year overall survival | Univariate Cox PH Model | | | Multivariable Cox PH Model | | |
| --- | --- | --- | --- | --- | --- | --- |
| Variable | p | Hazard Ratio | 95% CI | p | Hazard Ratio | 95% CI |
| Periodontitis  (periodontitis vs no periodontitis) | <0.001^*^ | 1.882 | 1.274 – 2.782 | 0.019^*^ | 1.613 | 1.082 – 2.406 |
| Sex  (male vs female) | 0.004^*^ | 2.009 | 1.186 – 3.403 | 0.005^*^ | 2.153 | 1.266 – 3.662 |
| Age at Surgery  (over 65 vs under 65) | 0.066^*^ | 1.306 | 0.982 – 1.738 | 0.059^*^ | 1.328 | 0.989 – 1.782 |
| Brinkman Index  (over 400 vs other) | 0.012 | 1.506 | 1.085 – 2.091 |  |  |  |
| Alcohol consumption  (Habitual vs other) | 0.057 | 1.426 | 0.975 – 2.084 |  |  |  |
| Tumor invasion (cT)  (T3-4 vs T1-2) | <0.001^*^ | 2.437 | 1.780 – 3.337 | <0.001^*^ | 2.608 | 1.585 – 4.294 |
| Lymph node metastasis (cN)  (N+ vs N0) | <0.001^*^ | 1.645 | 1.229 – 2.201 | 0.837 | 1.068 | 0.569 – 2.004 |
| Clinical Stage (UICC7)  (over 3A vs under 2B) | <0.001^*^ | 1.743 | 1.311 – 2.316 | 0.339 | 0.738 | 0.364 – 1.497 |
| Neoadjuvant therapy  (none vs with) | 0.560 | 0.919 | 0.691 – 1.222 |  |  |  |
| Tumor invasion (pT)  (T3-4 vs T1-2) | <0.001^*^ | 1.964 | 1.479 – 2.608 | 0.064 | 0.672 | 0.441 – 1.024 |
| Lymph node metastasis (pN)  (N+ vs N0) | <0.001^*^ | 1.857 | 1.400 – 2.465 | 0.683 | 0.916 | 0.613 – 1.459 |
| Pathological stage (UICC7)  (over 3A vs under 2B) | <0.001^*^ | 2.898 | 2.170 – 3.871 | <0.001^*^ | 2.843 | 1.700 – 4.754 |
| Albumin  (less than 4.0 mg/dl vs normal) | 0.010 | 1.273 | 0.956 – 1.694 |  |  |  |
| %VC  (under 80% vs other) | 0.231^*^ | 1.483 | 0.806 – 2.728 | 0.134^*^ | 1.614 | 0.863 – 3.018 |
| FEV1.0%  (other vs under 70% other) | 0.364 | 0.846 | 0.587 – 1.221 |  |  |  |
| Operation type  (Transthoracic vs Thoracoscopic) | <0.001^*^ | 1.725 | 1.264 – 2.354 | 0.151 | 0.783 | 0.914 – 1.786 |
| Operation time  (under 538 min vs over 538 min) | 0.515 | 0910 | 0.685 – 1.209 |  |  |  |
| Operation bleeding  (over 692 ml vs other) | 0.366 | 1.154 | 0.848 – 1.569 |  |  |  |
