# Supplementary material for: Pretreatment periodontitis is predictive of a poorer prognosis after esophagectomy for esophageal cancer
Source: Esophagus. 2024 Feb 20;21(2):120–30. doi: 10.1007/s10388-024-01045-z (PMC10957679; doi:10.1007/s10388-024-01045-z)
Supplement: Supplementary file 2 — Supplementary file2 (DOCX 22 KB) [file 10388_2024_1045_MOESM2_ESM.docx]

Supplementary Table 2

| 10-year Disease Specific survival | Univariate Cox PH Model | | | Multivariable Cox PH Model | | | | |
| --- | --- | --- | --- | --- | --- | --- | --- | --- |
| Variable | p | Hazard Ratio | 95% CI | | p | Hazard Ratio | 95% CI |  |
| Periodontitis  (periodontitis vs no periodontitis) | <0.001^*^ | 2.953 | 1.537 – 5.674 | | 0.022^*^ | 2.177 | 1.121 – 4.228 |  |
| Sex  (male vs female) | 0.009^*^ | 2.603 | 1.141 – 5.939 | | 0.019^*^ | 2.725 | 1.178 – 6.306 |  |
| Age at Surgery  (over 65 vs under 65) | 0.250^*^ | 0.797 | 0.540 – 1.174 | | 0.339^*^ | 0.822 | 0.550 – 1.228 |  |
| Brinkman Index  (over 400 vs other) | 0.045 | 1.575 | 0.994 – 2.498 | |  |  |  |  |
| Alcohol consumption  (Habitual vs other) | 0.081 | 1.589 | 0.919 – 2.748 | |  |  |  |  |
| Tumor invasion (cT)  (T3-4 vs T1-2) | <0.001^*^ | 4.771 | 2.797 – 8.140 | | 0.002^*^ | 3.965 | 1.663 – 9.454 |  |
| Lymph node metastasis (cN)  (N+ vs N0) | <0.001^*^ | 3.217 | 2.037 – 5.081 | | 0.077 | 2.475 | 0.906 – 6.765 |  |
| Clinical Stage (UICC7)  (over 3A vs under 2B) | <0.001^*^ | 3.116 | 2.044 – 4.749 | | 0.127 | 0.424 | 0.141 – 1.278 |  |
| Neoadjuvant therapy  (none vs with) | 0.128 | 1.366 | 0.909 – 2.054 | |  |  |  |  |
| Tumor invasion (pT)  (T3-4 vs T1-2) | <0.001^*^ | 3.377 | 2.266 – 5.034 | | 0.431 | 0.790 | 0.439 – 1.421 |  |
| Lymph node metastasis (pN)  (N+ vs N0) | <0.001^*^ | 3.945 | 2.578 – 6.037 | | 0.665 | 1.157 | 0.599 – 2.234 |  |
| Pathological stage (UICC7)  (over 3A vs under 2B) | <0.001^*^ | 6.188 | 4.156 – 9.213 | | <0.001^*^ | 4.147 | 2.002 – 8.591 |  |
| Albumin  (less than 4.0 mg/dl vs normal) | 0.835 | 1.042 | 0.705 – 1.541 | |  |  |  |  |
| %VC  (under 80% vs other) | 0.861 | 0.916 | 0.337 – 2.489 | | 0.537 | 1.384 | 0.493 – 3.884 |  |
| FEV1.0%  (other vs under 70% other) | 0.190 | 0.710 | 0.416 – 1.210 | |  |  |  |  |
| Operation type  (Transthoracic vs Thoracoscopic) | 0.001^*^ | 1.944 | 1.270– 2.976 | | 0.802 | 1.060 | 0.670 – 1.439 |  |
| Operation time  (under 538 min vs over 538 min) | 0.618 | 0.906 | 0.613 – 1.336 | |  |  |  |  |
| Operation bleeding  (over 692 ml vs other) | 0.524 | 1.150 | 0.751 – 1.761 | |  |  |  |  |
